# Cervical porcupine quill foreign body involving the spinal cord of a dog: A description of various imaging modality findings

**DOI:** 10.4102/jsava.v88i0.1549

**Published:** 2017-12-08

**Authors:** Christelle le Roux, Frans J. Venter, Robert M. Kirberger

**Affiliations:** 1 Department of Companion Animal Clinical Studies, University of Pretoria, South Africa

## Abstract

Although porcupine quill injuries are common in dogs, the detailed appearance of the quill on diagnostic ultrasound, computed tomography, and magnetic resonance imaging has not been sufficiently described. A 4-year-old, intact, female Jack Russel terrier presented with severe neck pain and ataxia after an altercation with a porcupine 2 weeks earlier. Radiology, diagnostic ultrasound, computed tomography and magnetic resonance imaging were all utilised to identify a quill imbedded in the cervical vertebral canal and cervical musculature and were compared to each other. Surgical removal of the quill, guided by imaging findings, led to the resolution of the clinical signs in the patient. Previous ultrasound imaging reports have just stated that the quill consists of paralell hyperechoic lines, and do not mention the finer hyperechoic lines inbetween and do not try to provide a reason for the appearance. Previous computed tomography (CT) reports just mention identifying the quill on CT images (whether or not CT could identify the fragments), but do not go into detail about the attenuating appearance of the quill nor try to relate this to the composition of the quill. This is to the authors’ knowledge the first report with detailed imaging descriptions of a case of cranial cervical vertebral canal porcupine quill foreign body in a dog. This is also the first report to allude to a possible difference in imaging findings related to quill structure because of keratin orientation and melanin content. The ideal imaging modality to use remains elusive, but ultrasound, computed tomography and magnetic resonance imaging could all identify the quill.

## Introduction

Porcupine quills, as well as wooden splinters, are common foreign bodies found in the canine head, especially the face, in North America (Grahn et al. 1995; Yanofsky, Bonneau & Breton 1986). Quill injuries caused by the African porcupine, *Hystrix africaeaustralis*, are not unusual in southern Africa. Quill migrations are common, but migration into the central nervous system is rare (Daoust 1991; Johnson et al. 2006; Schneider, Chen & Tucker 2010). Both septic and sterile foreign body reactions can result from penetrating porcupine quills, due primarily to their being sharp, barbed (North American or *Erethizon* spp.) or ridged or scaled (African or *Hystrix* spp.), which aids migration as well as being an irritant, and their often contaminated nature (Grahn et al. 1995; Sauvé, Sereda & Sereda 2012; Vincent & Owers 1986). An increased time between quill injury and presentation to the veterinarian is associated with an increased risk of complications; dogs that were presented for quill injuries more than 24 h after injury were 5.2 times more likely to experience complications, compared to dogs presented within less than 12 h (Johnson et al. 2006). Quills frequently break off, thus hampering their identification at the time of patient presentation for veterinary care (Guevara et al. 2015). Therefore, early diagnosis and subsequent complete removal improve the patient’s prognosis (Johnson et al. 2006). Determining the best diagnostic imaging modality to locate the quill is thus essential.

Several imaging modalities are available to the practitioner for identification of foreign bodies, including fistulography (Yanofsky et al. 1986), diagnostic ultrasound (US) (Brisson, Bersenas & Etue 2004; Grahn et al. 1995), computed tomography (CT) (Leskovar et al. 2003; Sauvé et al. 2012) and magnetic resonance imaging (MRI) (Schneider et al. 2010). However, few have described the quill itself in much detail.

This is the first report, to the authors’ knowledge, comparing all of the diagnostic imaging modality findings in a case of cranial cervical spinal cord penetration via the pharynx by a porcupine quill in a dog. This is also the first report to allude to a possible difference in imaging findings related to quill structure.

## Case presentation

A 4-year-old intact female Jack Russel terrier was referred with complaints of severe neck pain and ataxia. The patient had an altercation with a porcupine 2 weeks previously, and at that time several quills were removed from her mouth and around the mandible by the referring veterinarian. Of these quills, one had penetrated the soft palate and two more were found imbedded in the oropharynx. The owner stated that since the incident, the patient was at times so painful that she would only move her eyes. The patient presented clinically with ataxia and weakness with delayed proprioception of the right thoracic and pelvic limbs. Manipulation of the neck was painful in all directions, especially to the right side. Neuro-localisation was a right-sided lesion between C1 and C5. All other clinical parameters, routine haematology, serum biochemistry and urinalysis were within normal limits at presentation and during the stay in our hospital.

## Management and outcome

Supportive and pain therapy was instituted with maintenance intravenous fluid therapy (Ringer-lactate solution, Fresenius Kabi) at 18.3 mL/h, an intravenous constant rate infusion of fentanyl (Fentanyl 500 μg/10 mL, Pharma-Q Holdings [Pty] Ltd) at 3 μg/kg, and diazepam (Pax 10 mg/2 mL, Pharmacare Limited) intravenously at 0.5 mg/kg every 8 h. Gabapentin (Neurontin, Pfizer) and carprofen (Rimadyl, Zoetis) were added because pain control was not adequate initially (10 mg/kg per os every 12 h, and at 4.4 mg/kg once daily subcutaneously, respectively). The diazepam was concurrently decreased to 0.2 mg/kg intravenously every 4 h.

**FIGURE 1:**
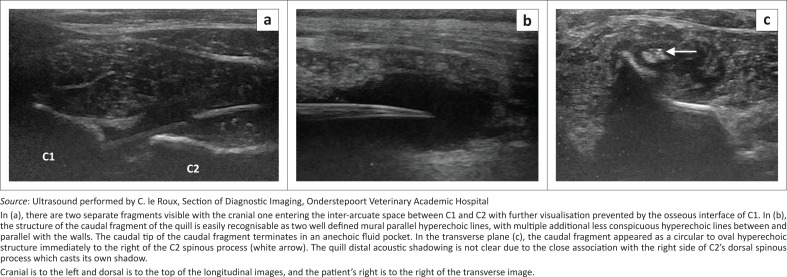
Dorsal longitudinal cervical soft tissue ultrasound performed at the levels of C1 and C2, (a) immediately right of the midline, (b) on the midline and (c) transverse image acquired over C2.

Orthogonal cervical radiographs were within normal limits. Soft tissue ultrasound was performed with a 10-MHz linear array transducer (Aloka ProSound F75, Hitachi-Aloka Medical Systems, Japan), over the dorsal cranial cervical region, and revealed what appeared to be two porcupine quill fragments (Figure 1), which in a longitudinal plane consisted of two well-defined mural parallel hyperechoic lines, with multiple additional less-conspicuous hyperechoic lines between and parallel with the walls. In the transverse plane, they appeared as a circular to oval hyperechoic structure with distal acoustic shadowing. The first and more cranial quill fragment was approximately 1 mm wide by 12 mm long and was located dorso-laterally to the lamina of C2, slightly to the right of the midline, with a blunt caudally directed end and its cranially directed end in a cranio-ventral direction and thus not visualised because of the bony interface of the C1 lamina superficial to it. The second fragment, in a similar orientation and 3.7 mm wide and approximately 55 mm long, was visualised immediately caudal to the first over the lamina of C2 and C3, with its pointed end caudally directed towards the midline, and located within an anechoic fluid pocket. Both fragments were surrounded by a poorly marginated hyperechoic rim with anechoic to mildly hypoechoic fluid centrally, within heterogeneous mixed hypo- and hyperechoic subcutaneous tissue. This was believed to be exudative fluid, such as a chronic seroma or haemorrhage, with cellulitis. The lack of echogenic specks within the anechoic fluid made an abscess unlikely.

**FIGURE 2:**
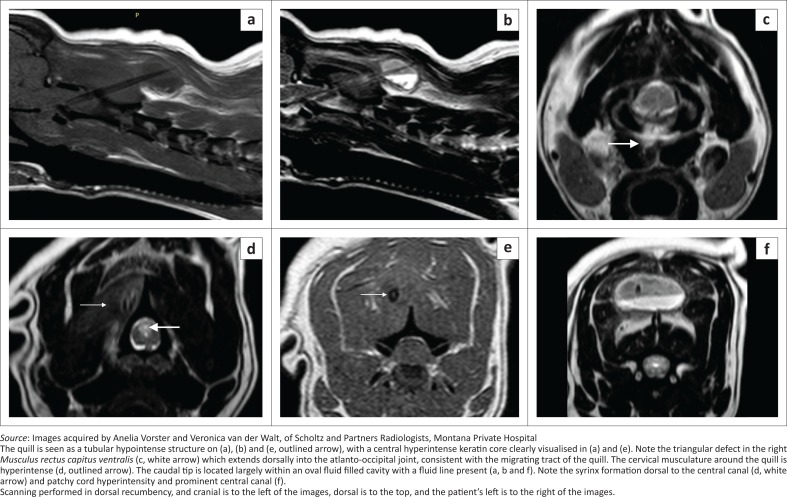
(a) Sagittal T1, (b) T2-weighted, (c, d, and f) transverse T2-weighted and (e) T1-weighted magnetic resonance images of the cervical spine. Image (c) is at the level of the atlanto-occipital joint, (d) at cranial C2, (e) at C2–3 and (f) at C3.

An MRI study in dorsal recumbency was performed the following day (Philips Achieva 1.5T, South Africa), to visualise the cranial cervical spinal cord and surrounding tissues. Only pre-contrast images were obtained as the administration of contrast at that time was not deemed necessary (Figure 2a–f).

The quill was visualised as a cranio-ventral to caudo-dorsal orientated T2-weighted (T2W) and T1W hypointense (similar to the cortices of the vertebrae), single 66 mm long × 4.6 mm wide tubular structure, with a 1-mm hyperintense central core (isointense to muscle) (Figure 2a, b and e). The quill appeared better delineated on the T1W sequence compared to the T2W sequence. The quill walls converged caudally to a point in the dorsal cervical musculature at the level of caudal C3 remaining to the right of the midline, and cranially entered the C1–2 inter-arcuate space slightly to the right side, where it appeared to fray or fragment into 5–7 small fragments caudo-dorsally to the dens with a single fragment extending more cranial than the rest but still in a tubular orientation. The spinal cord at the level of the dens was displaced and compressed towards the left because of the quill being located in the right extramedullary space and a focus of T2W hyperintense and T1W isointense poorly defined material, believed to be granulomatous tissue, located lateral to the cord and the quill. The right half of the cord was mildly T2W hyperintense, consistent with oedema or gliosis. Ventro-laterally to the cord on the right side, there was a curved focal T2W, T1W and gradient echo hypoattenuating 6 × 2.3 mm focus that was compatible with focal haemorrhage 14 days previously. The central canal was mildly distended with dorsal syrinx formation, up to caudal C4, with moderate cord swelling (Figure 2d). The right *Musculus rectus capitus ventralis*, from its origin on the skull caudally, had an incomplete sagittal T2W hyperintense tract within it, with hyperintensity of the rest of the muscle adjacent to the tract. At the level of the atlanto-occipital joint, there was a T2W triangular defect in the muscle that was continuous with the joint space dorsally; thus, penetration and synovitis of the joint were suspected (Figure 2c). This defect region extended dorsally into the vertebral canal, ventrally and laterally to the dens. The dorsal cervical muscles, in which the quill was embedded, were T2W hyperintense, consistent with oedema and inflammation (Figure 2d). The caudal third of the quill, except for its caudal tip, was surrounded by a well-defined structure within which was a clearly defined horizontal fluid line; the non-dependent half was T2W hyperintense and T1W hypointense and the dependent half was T2W hypointense and T1W isointense to muscle (Figure 2a, b and f). This was consistent with sedimented proteinaceous fluid, for example, a seroma, haematoma or less likely, an abscess. It was surrounded by a thin peripheral T2W hypointense and T1W hyperintense rim, consistent with a proteinaceous tissue rim. The MRI diagnosis was a single broken-off porcupine quill, with penetrating tract from the roof of the nasopharynx via the atlanto-occipital joint space into the vertebral canal exiting via the C1–C2 inter-arcuate space and ending in the dorsal cervical muscles.

**FIGURE 3:**
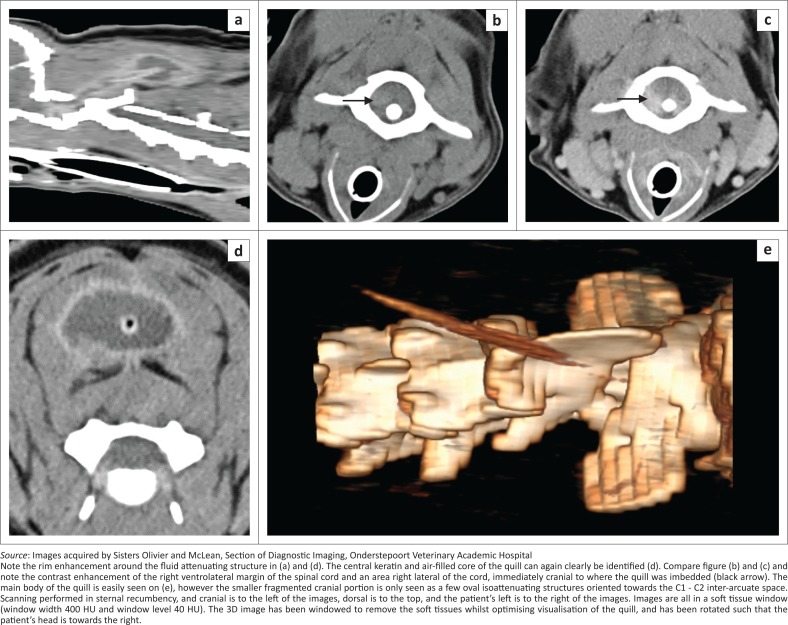
(a) Sagittal post-contrast, (b) transverse pre-contrast and (c, d) transverse post-contrast computed tomography images, at the level of the middle of C1 (b and c) and at the level of C3 (d). Volume rendered 3-dimensional reconstruction of the cranial cervical spine (e).

Pre- and post-contrast helical CT (Somatom Emotion Duo, Siemens Healthcare, Forchheim, Germany) was performed the next day, immediately pre-operatively, to compare the findings with that of MRI. A soft tissue window (window width of 350 Hounsfield units [HU] and window level of 40 HU) was used with 2-mm slice thickness and 50% reconstruction increments. For the post-contrast scan, iohexol (Omnipaque 300 mg I/mL, GE Healthcare [Pty] Ltd) was administered intravenously at 1.5 mL/kg and the scan commenced 2 min after the end of injection. A linear mildly hyperattenuating (HU of 115–160) quill was seen extending dorso-caudally in the same orientation as seen on the MRI (Figure 3a). However, differing from the MRI, there appeared to be a thinner 1.5 mm wide by 18.2 mm long cranially located fragment, which was closely associated with a 4.8 mm wide by 66 mm long caudally located fragment, with only 1 mm – 2 mm space between the two. The mid- to caudal portion of the caudal fragment had a hypoattenuating (mean HU −215) central core of 3.2 mm wide (Figure 3d). The mid- to caudal portion of the quill was contained within a hypoattenuating (HU 20) oval structure that demonstrated marked ring enhancement post-contrast (HU of rim 130), which corresponded to the suspected haematoma or seroma seen on MRI and US (Figure 3a and d). The caudal fragment extended further caudally than seen on MRI, up to caudal C4 and to the left of midline, which indicated some caudal migration of the fragment between the studies. Adjacent to the cranial tip of the quill, and somewhat obscuring it, was poorly defined hyperattenuating focus (HU 60–90) that demonstrated moderate heterogeneous contrast enhancement (125 HU) within the right ventro-lateral spinal cord as well as the area adjacent to the cord, suspected to be because of neovascularisation and corresponding to the area of granulation tissue and haemorrhage suspected on MRI (Figure 3b and c). The musculature around the quill had a mildly heterogeneous appearance with a hypoattenuating rim around the quill (HU 30) and showed mild contrast enhancement (HU 100 vs. 67 pre-contrast), similar to the T2W appearance seen on MRI. The signal abnormalities of the *M. rectus capitus ventralis*, as noted on MRI, were not seen initially, but on retrospective viewing a poorly defined region of subtle irregular contrast enhancement (HU 95) was noted with a triangular less attenuating focus, believed to be the same presumed tract seen on MRI. Overall, the CT and MRI findings correlated well. However, with CT, less evidence for a migratory tract was identified, and spinal cord changes were greatly underestimated. Computed tomography was highly suggestive of two fragments, which were not noted on MRI, with the caudal migration of the quill suspected.

**FIGURE 4:**
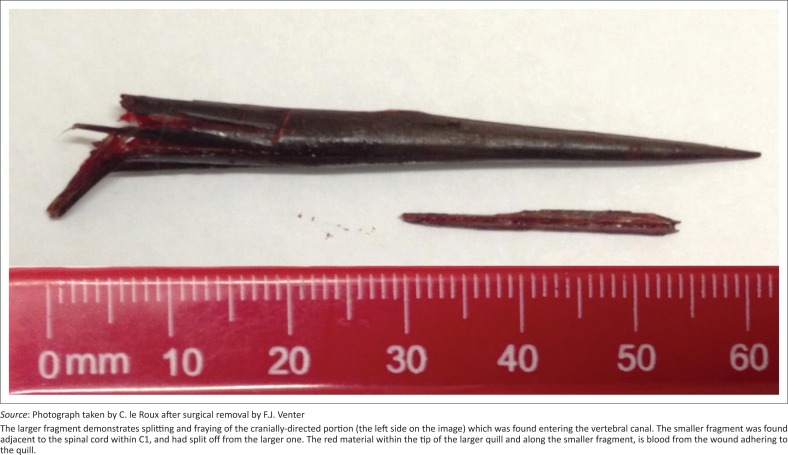
Photograph taken after surgical removal of the porcupine quill.

A dorsal midline incision was made that extended from the external occipital protuberance to the level of C4, exposing the nuchal ligament. The right dorsolateral cervical muscles were separated after an incision through the median fibrous raphe, and the right *M. rectus capitus, M. spinalis et semispinalis cervicis* and *Mm. multifidus* were incised to expose the right side of the spinous processes. Within the abovementioned musculature, a pocket of fluid was identified with the 60-mm long quill fragment embedded in a caudo-dorsal direction. The quill was removed. A second, shorter fragment, 25 mm long, was identified entering the spinal canal at the level of the C1–C2 inter-arcuate space and was surgically removed after a partial dorsal laminectomy was performed (Figure 4). This fragment did not penetrate the cord and the two fragments were separated from one another but appeared to originate from the same quill.

The patient improved daily during 4 days’ post-operative hospitalisation and at discharge on the 4th day demonstrated mild ataxia even though neck guarding was still present. The patient had good neck mobility in all directions.

## Ethical considerations

This is a retrospective case report describing a client-owned dog using data retrieved and reviewed from clinical records of our academic hospital. The dog, during its period of hospitalisation, was treated and housed according to standard hospital protocols for the management of client-owned pets. No tests or treatments were conducted for research purposes. The owner provided standard signed consent for the data and images to be used for publication.

## Discussion

The route by which migrating foreign bodies penetrate the central nervous system is often unclear (Dennis et al. 2005). Migrating tracts may not be evident, and thus it has been speculated that foreign material may perforate the nasopharyngeal or pharyngeal soft tissues and enter the cervical vertebral canal via the atlanto-occipital joint (Brockman & Trout 1991; Leskovar et al. 2003). This is supported in our patient, as a suspected migrating tract was identified on MRI that communicated with the atlanto-occipital joint space. Quills had also been removed from the oral cavity by the referring veterinarian, leaving behind the caudal intact point of the quill, which was directed caudally into the cervical muscles. In a CT case report of porcupine quill migration into the left cerebral hemisphere of a dog, the quill’s direction and proximity to the left foramen ovale led the authors to believe that migration was via the nasopharynx (Sauvé et al. 2012).

Radiographs are of little benefit in identifying foreign bodies such as plant material and wood or porcupine quills (Grahn et al. 1995; Schneider et al. 2010; Stander & Kirberger 2011; Yanofsky et al. 1986). Fistulography or sinography, a technique in which iodinated contrast medium is injected into a fistula or draining sinus, may potentially identify foreign objects if the object creates a filling defect in the contrast (Yanofsky et al. 1986). This technique is dependent on the presence of a cutaneous sinus tract; hence, it was not applicable in our patient.

Ultrasound findings in our report correlate well with literature reports (Grahn et al. 1995; MacKay & Mattoon 2015). In our case, the quill had multiple additional less-conspicuous hyperechoic lines parallel within the walls of the quill, which have not been described. This is speculated by the authors to be because of a difference in quill architecture in the African porcupine, compared to the North American species described in previous ultrasound reports. The porcupine quill is composed entirely of keratin and consists of an outer sheath called the cortex and a porous core called the foam (Yang, Chao & McKittrick 2013). However, *Hystrix* spp. have solid longitudinal keratin ‘stiffeners’ within the quill core that converge from the cortex towards the centre (Vincent & Owers 1986; Yang et al. 2013), giving the quill the appearance of an ‘orange-slice’ on scanning electron microscopy (Vincent & Owers 1986). The authors believe these radial keratin structures are what could be seen as multiple hyperechoic lines within the quill. Echocardiography appears to be a good modality for detecting intra-cardiac migratory quills, but few cases have been described (Costa et al. 2014; Guevara et al. 2015; Nucci & Liptak 2016). Ultrasound imaging is obviously limited by bone or air interfaces (Mattoon & Nyland 2015), with further visualisation of the cranial quill in our case not being possible, despite being suggestive of vertebral canal location. Another limitation that the senior author found was the difficulty in identifying the quill in the cervical muscle as a single quill, because of the two-dimensional nature of US scanning and the limited size of the probe footprint, which, in this case, could not scan more than 35 mm length at a time.

The use of MRI has only been reported twice in the diagnosis of a quill foreign body in a dog (Flesher, Lam & Donovan 2017; Schneider et al. 2010). The quill was described in one case as a circular well-defined T2W hypointense structure that had a similar appearance to the basilar artery and was thus only identified retrospectively after an exploratory laminectomy. Our case was similar in appearance, but a central core was also found, which was likely because of the main body of the quill also being embedded and not just the tip. The appearance of the core is likely because of the differential structure of the keratin that makes up the wall versus the central portions of the quill (Yang et al. 2013). In the second report, an MRI of the cervical region failed to directly visualise the quills, with only suspected migratory tracts visualised (Flesher et al. 2017).

The use of CT clearly demonstrated the quill in this case and was in the authors’ opinion superior to demonstrate the quill, contradictory to some of the literature. A case of an intra-cranial porcupine quill described the HU as ranging from 52 to 68 (Sauvé et al. 2012), which is markedly lower than 115–160 as found in our study. Another report on thoracic, including intra-cardiac, migration of quills, reported that they exhibited a soft tissue attenuation (HU not given) that made detection difficult, and thus a quill within a cardiac chamber was missed (Guevara et al. 2015). A third report (Costa et al. 2014) also could not detect an intra-cardiac quill. Although not mentioned in any of the three studies, it would appear from all the post-mortem images of the quills that they were unpigmented, whilst our quill was entirely black, and it would be interesting to speculate whether the pigments could alter the measured HU. Quills are composed totally of keratin (Yang et al. 2013), and melanin is the pigment responsible for its colouring (Rose 2012b). In a recent study of melanomas in the equine head (Dixon et al. 2016), the median pre-contrast HU value of the neoplastic masses was 113.5, and the hyperattenuation identified was believed to be as a result of intracytoplasmic melanin pigment. This may explain the differences between the quills’ HU in the previous reports; in that our quill was black. In two other reports, one of ventricular myocardial migration (Nucci & Liptak 2016) and another of head and thoracic migration (Flesher et al. 2017), CT failed to detect the quills. Reasons for non-visualisation were given as location of the foreign body, slice thickness (5 mm), lack of contrast administration, and experience of the radiologist (Nucci & Liptak 2016). An obvious disadvantage with quill detection in thoracic CT is motion artefact, which was not the case in our dog.

Quills are reported to contain air (Rose 2012a), but the appearance of air was not typically found in any imaging modality. Ultrasound did not have any reverberation artefact typical of gas, and the far wall of the quill was easily seen, which would not be the case if a significant air core was present. The CT attenuation was consistent with some air content (−215) when compared to the tracheal air (−1000), but this was believed to be as a result of averaging of the keratin foam core and the air (HU of −1000) between the keratin components. This is also consistent with the appearance of a central soft tissue intensity seen on the MRI study, rather than hypointensity to signal void, which would be consistent with air. The other likely possibility is that, because of the broken-off nature of the quill and its chronicity, some haemorrhage or inflammatory fluid may have entered the core and thus greatly decreased or obliterated the expected gas content.

It is unclear as to why the quill in our case was so easily and completely identified compared with previous reports, but because *Hystrix* spp. quills are longer and also of larger diameter than those of *Erethizon* spp. (Yang et al. 2013), this may be the most important reason. Other reasons may include the superficial anatomical location, lack of motion artefact in the spine, the large size of the fragment, contrast differences compared to the surrounding tissues (possibly because of the melanin pigment), and possibly imaging modality (spatial and contrast resolution, etc.) and operator factors. Intravenous contrast administration was not felt to contribute much information to the visualisation of the quill itself but benefits the detection of additional soft tissue changes and may help identify migratory tracts if the quill itself cannot be found. The small measured size differences of the quill between the modalities were felt to be negligible, and many external factors could have played a role (mainly modality type and operator). It is interesting that US and CT correlated best to the number of fragments detected, and it was believed that the intensity of the quill and the suspected haemorrhage, being similar, may have led to the quill being obscured on MRI. Also, MRI has poorer spatial resolution than ultrasound and CT, despite its excellent contrast resolution, and this may also have played a role.

## Conclusion

This is, to the authors’ knowledge, the first report with detailed diagnostic imaging descriptions in a case of a porcupine quill foreign body in a dog. This is also the first report to allude to a possible difference in imaging findings related to quill structure because of keratin orientation and melanin content. The ideal imaging modality to use remains elusive, but US, CT and MRI could all identify the quill. However, diagnostic ultrasound should be the first imaging modality used because of being readily available in practice and low cost.
